# Clinical Reasoning Skills Among Second-Phase Medical Students in West Bengal, India: An Exploratory Study

**DOI:** 10.7759/cureus.68839

**Published:** 2024-09-06

**Authors:** Diptakanti Mukhopadhyay, Sonali G Choudhari

**Affiliations:** 1 Community Medicine, College of Medicine & Sagore Dutta Hospital, Kolkata, IND; 2 Community Medicine, School of Epidemiology and Public Health, Jawaharlal Nehru Medical College, Datta Meghe Institute of Higher Education & Research, Wardha, IND

**Keywords:** clinical case management, diagnostic skills, key feature questions, medical student assessment, undergraduate medical education

## Abstract

Introduction

Proper application of clinical reasoning skills is essential to reduce diagnostic and management errors. Explicit inclusion of training and assessment of clinical reasoning skills is the demand of time. The study intended to measure the clinical reasoning skills of second-phase undergraduate students in a medical college in West Bengal, India, and its distribution across several individual variables.

Methods

The clinical reasoning skills of 105 undergraduate medical students were assessed in a cross-sectional exploratory study using key feature questions (KFQs) with the partial credit scoring system. Six case vignettes aligned to the core competencies in the subject of pharmacology, pathology, and microbiology were designed and validated by the subject material experts for this purpose. The responses of the participants were collected through Google Forms (Google, Mountain View, CA) after obtaining written informed consent. The scores obtained in all KFQs were added and expressed in percentage of the maximum attainable score.

Results

The mean (±SD) clinical reasoning score of the participants was 42.5 (±12.6). Only 29.6% of respondents scored ≥ 50. Students with higher subjective economic status (p-value = 0.01) and perceived autonomy (p-value < 0.001) were more likely to have higher clinical reasoning scores. The marks obtained in previous summative examinations were significantly correlated with clinical reasoning scores.

Conclusion

Average score < 50.0 and inability to score ≥ 50.0 by more than two-thirds of the participants reflected the deficit in the clinical reasoning skills of second-phase MBBS students. The association of clinical reasoning skills with economic status, autonomy, and previous academic performances needs further exploration.

## Introduction

Clinical decision-making often involves significant uncertainty, particularly in defining diseases, evaluating treatment outcomes, and making management decisions [1]. Doctors must make informed decisions based on patient narratives, physical examinations, and relevant tests. Poor decision-making in these situations can compromise patient outcomes and lead to adverse incidents, which can negatively impact the doctor's reputation and the standing of the healthcare institution [2]. Most diagnostic errors originate from mistakes in clinical reasoning [3]. Additionally, numerous errors in medical management were associated with the incorrect application of established therapeutic guidelines, including underutilization, overutilization, or misuse [3,4]. Studies have shown that even experienced clinicians are likely to make mistakes due to lapses in reasoning rather than a lack of knowledge [5].

Clinical reasoning was described as a complex process involving the identification of clinical data, its prioritization through detailed analysis to form a hypothesis, and the creation of a plan to test and either confirm or refute that hypothesis [6]. Cognitive processing skills and knowledge are interconnected and enhanced cognitive and metacognitive abilities can improve conceptual understanding and problem-solving skills even in unfamiliar contexts [3,7]. Physicians must be aware of how personal views, beliefs, and biases influence their decision-making [8,9]. By engaging in cognitive and metacognitive processes, clinicians can improve diagnostic and management outcomes for patients [3,8]. Linn proposed that clinical reasoning skills were evident in every clinical interaction [10]. During history taking, physicians could discern critical information and formulate several hypotheses, which were then further refined through physical examination. These hypotheses were later confirmed or refuted through laboratory investigations [10].

Although the importance of developing clinical reasoning skills in undergraduate medical students is frequently emphasized, there is a notable scarcity of empirical studies on this topic, especially in India, even after a thorough search of academic databases.

This study intended to measure the clinical reasoning skills of second-phase Bachelor of Medicine and Bachelor of Surgery (MBBS) students and its distribution across selected individual variables in a medical college in Kolkata, West Bengal.

## Materials and methods

An exploratory, cross-sectional, descriptive study was conducted between May and July 2024 among second-phase students enrolled at a government medical college located on the outskirts of Kolkata, West Bengal, India. The total sample size was determined to be 106 using the OpenEpi program [11]. This was founded on the notion that at least half of the students would perform better than the median on the clinical reasoning test, with a 95% confidence level, a 10% absolute precision rate, and a 10% non-response rate. The 106 students were selected through simple random sampling from the list of roll numbers of the second-phase MBBS students.

Clinical reasoning skills were assessed using six case scenario-based key feature questions (KFQs), two each from the core competencies under pharmacology, pathology, and microbiology. A series of KFQs was designed and validated, involving subject material experts from the concerned disciplines and medical education. The objective marking pattern and model answers were also validated. Each KFQ was allotted five marks. The case scenarios and follow-up questions involved the identification of necessary information, organization, and logical analysis, followed by decision-making on diagnosis and/or management. The cases and questions were carefully selected from the topics of the curriculum that the students had already learned. The marks scored in each of the six questions were added to obtain the total score, and it was standardized by expressing it as a percentage of the maximum achievable score. The higher the score, the higher the clinical reasoning skills.

Gender, subjective economic status, perceived family support, perceived autonomy, marks in the higher secondary or equivalent examination, and first professional MBBS examination were the independent variables. The students were asked to rate their subjective economic status on a scale of very well-off, living comfortably, just getting by, almost poor, and poor [12]. For analysis, the scale was dichotomized where the last three categories were collated to form a category "economically weaker" while the rest of the categories were clubbed together to form a new category "economically well-off." The participants were asked to rate their autonomy in making decisions about academic activities, leisure time activities, and social interaction, according to their perception on a five-point scale: very much, moderate, neutral/not to comment, less, and very less. Similarly, the participants were asked to rate their family support including economic, emotional, and social support from the family and family members, according to their perception on a five-point scale: very much, moderate, neutral/not to comment, less, and very less. The perceived family support and perceived autonomy were recategorized, clubbing the last three categories into "low" and the first two categories as "high."

An anonymous, structured questionnaire was distributed to the selected students via their WhatsApp (Meta, Menlo Park, CA) numbers using Google Forms (Google, Mountain View, CA). The initial section included an informed consent form, which participants needed to complete before proceeding to the subsequent sections. Students were asked to submit their responses within three days of receiving the questionnaire.

The responses in Google Forms were exported into a Microsoft Excel spreadsheet (Microsoft Corporation, Redmond, WA) and checked for consistency. In this study, electronic data capturing was used. So, the chance of human error in entering data was almost non-existent. So, checking consistency involves the process of checking whether the format of the data conforms to the data record format, any outlier, or any missing value. The summary measure with the dispersion of the clinical reasoning score was expressed using the median (IQR) and mean (±SD). Mann-Whitney U test was used to check the difference in scores according to different independent variables. Spearman's rho coefficient was used to assess the correlation of clinical reasoning scores with marks obtained in higher secondary or equivalent examinations as well as the first professional MBBS examination.

The study obtained approval from the Institutional Ethics Committee of the respective medical college (Registration No.: ECR/1210/Inst/WB/2019/RR-22) with reference number CMSDH/IEC/107/12-2023 (dated: 4 December 2023).

## Results

Out of the total, 106 students were approached by sending the link to Google Forms, and 105 completed forms were submitted. After consistency checking, analysis was conducted with the data from 105 participants.

The mean (SD) and median (IQR) ages of the participants were 22.0 (2.1) and 21.0 (3.0) years, respectively, with a range of 20-29 years. Among the study participants, 41 (39.0%) were females, and the rest were male students. As per subjective economic status, 11 (10.5%) were poor/almost poor, and 61 (58.1%) were from families living comfortably or very well-off. About family support, 95 (90.5%) perceived their family support as moderate or very much. Similarly, 90 (85.7%) study participants perceived their autonomy as moderate or very high. The average marks obtained in higher secondary or equivalent examination and first professional MBBS examination were 87.1% (±9.2) with a range from 61.0% to 99.0% and 58.3% (±6.0) with a range from 50.3% to 78%. Mean (±SD) and median (interquartile range) values of the clinical reasoning score of the participants were observed to be 42.5 (±12.6) and 43.3 (13.3), with 31 participants (29.5%) scoring 50.0 or above.

Table 1 indicated that there were no significant differences in clinical reasoning scores based on participants' gender or perceived family support. However, it was observed that females had a higher mean clinical reasoning score (43.7) compared to males (41.8), and participants with perceived low family support had a higher mean score (43.6) than those with perceived high family support (42.3). Additionally, the table also revealed that participants from economically well-off families had a significantly higher (Mann-Whitney U = 940; p-value = 0.010) clinical reasoning score (mean (±SD) = 45.6 (±11.3)) compared to those from economically weaker families (mean (±SD) = 38.1 (± 13.2)). Moreover, the clinical reasoning score was significantly higher (Mann-Whitney U = 270; p-value < 0.001) among participants with high perceived autonomy (mean (±SD) = 45.0 (±10.9)) than those with low perceived autonomy (mean (±SD) = 29.6 (±13.3)).

**Table 1:** Distribution of clinical reasoning score of the participants according to their gender, subjective economic status, perceived family support, and perceived autonomy.

Variables	Categories	Clinical reasoning score			Mann-Whitney U (p-value)
		No.	Mean (SD)	Median (IQR)	
Gender	Female	41	43.7 (12.8)	45.0 (15.0)	1179 (0.383)
	Male	64	41.8 (12.6)	43.3 (13.5)	
Subjective economic status	Weaker	43	38.1 (13.2)	41.7 (19.2)	940 (0.010)
	Well off	62	45.6 (11.3)	45.0 (16.7)	
Perceived family support	Low	16	43.6 (13.2)	43.3 (17.5)	663 (0.661)
	High	89	42.3 (12.6)	43.3 (13.3)	
Perceived autonomy	Low	17	29.6 (13.3)	30.0 (21.7)	270 (<0.001)
	High	88	45.0 (10.9)	45.0 (13.7)	
Total		105	42.5 (12.6)	43.3 (13.3)	-

Table 2 revealed that there was a significant correlation between clinical reasoning score and marks obtained in the first professional MBBS examination (Spearman’s ρ = 0.636; p-value < 0.001) and that of higher secondary or equivalent examination (Spearman’s ρ = 0.307; p-value = 0.001). The marks obtained in the first professional MBBS examination were also significantly correlated with marks of higher secondary or equivalent examination (Spearman’s ρ = 0.383; p-value < 0.001).

**Table 2:** Correlation between clinical reasoning score, marks in the last summative examination in the medical course, and marks in the higher secondary or equivalent examination.

Variables	Correlation coefficient	Marks of last summative examination in medical course	Marks of higher secondary or equivalent examination
Clinical reasoning score	Spearman’s ρ (p-value)	0.636 (<0.001)	0.307 (0.001)
Marks of last summative examination in medical course	Spearman’s ρ (p-value)	-	0.383 (<0.001)

## Discussion

Diagnoses and treatment plans are two major outcomes of clinical decision-making [13]. Moreover, the appropriateness of the second one is primarily dependent on the correctness of the first one. The errors in diagnoses and management as the result of cognitive failures can stem from the human data management and processing system [13]. It was reiterated in several studies that roughly, there were two modes of processing information - intuitive or analytic [14]. The intuitive process was reflexive and acquired through repeated exposures to similar clinical conditions [15]. In contrast, the analytic mode is slow, deliberate, and usually reliable, and it also follows the "laws of science and logic" [13].

Assessment of clinical reasoning skills is always challenging as those are not overtly observable. Different assessment methods starting from different types of multiple-choice questions (MCQs), modified essay-type questions, script concordance tests, key feature tests, long cases, oral examinations, direct observation methods, etc., were used to assess clinical reasoning skills [16,17]. It was reported that the assessment of clinical reasoning was context-specific for its dependence on related biomedical knowledge [18].

There are studies where different methods were used to assess clinical reasoning skills [19,20]. In the present study, KFQs from multiple areas were used to assess the clinical reasoning skills of second-phase MBBS students [16,21]. It was reported that KFQs were reliable and valid if properly designed using blueprinting [21]. The test based on KFQs could effectively measure different constructs of clinical reasoning skills, although more KFQs would increase the reliability of the test [16,21]. It was also observed to be used as a teaching aid to enhance the clinical decision-making skills of dental students [22].

It was observed that early inclusion of teaching and assessment of clinical reasoning skills might have long-term effects on the clinical decision-making of medical professionals [23]. It was also documented that the early introduction of the clinical reasoning curriculum increased their decision-making abilities during clerkship [24,25].

In the present study, it was observed that the average score of second-phase students was less than 50.0% on the entire scale, and less than one-third of students scored more or equal to 50.0%. The findings were corroborated by Rencic et al. [26]. It signified lapses in clinical reasoning. The questions were aligned with the core competencies of the curriculum and validated for content by several subject material experts. This deficit in performance might be explained either by a lack of reasoning skills or by the possessed biomedical knowledge. However, it needs further exploration.

There was no difference in clinical reasoning scores based on gender and perceived family support. Although research findings reported more intuitive decision-making by females, the present study reflected the reverse findings [27]. The students of economically well-off families performed significantly better than their counterparts [28]. It was observed by Pearl et al. that children having a quality environment in the household were better problem solvers [29]. The students with high perceived autonomy had higher clinical reasoning scores in the present study. It was observed that a democratic environment in the household and greater autonomy incited critical thinking skills, which, in turn, had influences on clinical reasoning [28]. When students were encouraged to take on more responsibilities, they became proactive in problem-solving [30].

The significant correlation of clinical reasoning scores with academic performances in higher secondary or equivalent examinations, as well as summative examination of the undergraduate medical course, underlined the association of knowledge components in clinical reasoning [18].

The present was among the first few studies on assessing the clinical reasoning skills of medical students in the para-clinical phase. It used a robust method to assess clinical reasoning using key feature tests. The key feature test had higher reliability and validity, which was further strengthened by simple language and partial credit scoring [21]. Clinical reasoning skills are of utmost importance to reduce diagnostic and management errors. A valid assessment system is a prerequisite for incorporating clinical reasoning explicitly as an element of the undergraduate curriculum. This study might pave the way in this regard. The tests were taken from all three subjects on different clinical scenarios and assessed by several subject material experts before administration. However, more KFQs would enhance the reliability of the test [21]. The small number of students from one medical college and six test questions might affect the reliability and generalizability of the study findings. The overall low clinical reasoning score among the participants and the distribution of clinical reasoning scores across the selected variables need further exploration.

## Conclusions

Clinical reasoning is the process of identifying the key features to develop probable hypotheses, supporting or refuting these through physical examination and relevant laboratory tests, determining treatment plans based on possibilities of positive outcomes, and refining those as and when needed. Although the starting point is the available standard guidelines and research findings, it generally goes beyond it through the synthesis of knowledge using higher-order cognitive skills based on the specific context. So, teaching and training those skills are necessary along with acquiring biomedical knowledge, preferably through problem-oriented methods.

The present study, one of the very few studies in an Indian setting, emphasized lapses in clinical reasoning skills of second-phase (pre-clerkship) medical students. However, ascertaining the factors for these lapses was beyond the scope of this study. Although gender and perceived family support were not significantly associated with clinical reasoning scores, the high subjective economic status and perceived autonomy were significantly associated with higher clinical reasoning scores. Students with higher academic performances were more likely to have higher clinical reasoning scores in this study.

## Appendices

Questionnaire for second-phase medical students

1. Age: ___________________ years

Please encircle the most appropriate answer:

2. Gender: Male/Female/Other/Not to disclose

3. How do you perceive the economic condition of your family?

Very well-off/Living comfortably/Just getting by/Almost poor/Poor

4. How do you perceive your family support, considering all aspects?

Very much/Moderate/Neutral/Less/Very less

5. How do you perceive your autonomy to make decisions, considering all aspects?

Very much/Moderate/Neutral/Less/Very less

Key Feature Questions

1) An eight-year-old girl presented to the pediatrician with a complaint of weakness and low-grade fever for one month. Her mother reported a few episodes of mucosal bleeding during that period. The girl was anemic with a hemoglobin level of 8.9 gm/dL. Peripheral blood smear examination showed a good number of large, round, mononuclear cells with deeply basophilic cytoplasm, whose exact nature could not be categorized morphologically.

a) What is the most probable diagnosis?

b) Enlist at least two features mentioned above to support the diagnosis.

c) What other clinical finding(s) may be looked for that may help diagnose the problem?

d) Propose at least two investigations to confirm the diagnosis.

2) A 52-year-old, male cab driver with a history of smoking for the last 30 years presented at the chest medicine OPD with a cough for three months, weight loss, worsening respiratory distress, and occasional low-grade fever. On examination, pallor (with hemoglobin = 8.9 gm%) and an enlarged right supra-clavicular lymph node were found. Chest X-ray showed a radio-opaque shadow in the right upper lobe with hilar lymphadenopathy.

a) Enlist three differential diagnoses based on the presenting features.

b) Suggest the most probable diagnosis based on clinical, radiological, and other features.

c) Name one investigation to confirm the diagnosis.

d) Name the most sensitive and specific investigation in this case to confirm the diagnosis.

3) A 20-year-old medical student comes with a complaint of examination-related anxiety and inability to sleep for the past seven days. She has been diagnosed with short-term insomnia.

a) Between diazepam, alprazolam, and zolpidem, which drug would be your choice for this student?

b) Cite at least one reason favoring your decision.

c) Which two key points should be communicated to the patient on the prescription of any of the above drugs?

d) On the prescription of alprazolam, the patient comes back to the doctor after one week with complaints of excessive drowsiness and daytime sleepiness. History reveals she has been on first-line anti-tubercular drugs for the past one month. Explain the connection.

4) A 52-year-old obese and diabetic corporate employee is diagnosed with habitual constipation.

a) Out of tablet bisacodyl, psyllium husk, and syrup lactulose, which one would benefit the patient most, according to you?

b) Cite at least two reasons for choosing the particular drug.

c) This patient develops acute upper abdominal pain and is scheduled for whole abdomen ultrasonography. In this case, which agent out of those three could be useful for bowel preparation?

d) Cite one reason to select the particular drug.

5) A 10-year-old child arrived in the emergency room with complaints of high-grade continuous fever with headache, myalgia, and abdominal pain for four days. He had two episodes of vomiting yesterday.

a) Enlist three other information you require to note in the history.

b) Enlist at least four important physical parameters you must check in this patient.

c) Mention the three most important investigations you would send to confirm the diagnosis.

6) A 24-year-old male Uber driver presented with a history of jaundice and elevated liver enzymes for the last six months. He received treatment including injections and oral tablets from a private nursing home after a road traffic accident eight months ago with a history of two units of blood transfusion. After serological investigation, the person was found to be positive for HBsAg (hepatitis B surface antigen), anti-HBC IgM, and HBeAg (hepatitis B e-antigen) and negative for anti-HBs and anti-HBe.

a) Interpret the result.

b) If the person was found to be positive for HBsAg, anti-HBC IgG, and anti-HBe and negative for anti-HBs and HBeAg, what would be your interpretation?

c) Based on case history, enlist the probable reason(s) for this infection.
